# Circulating biomarker correlates of left atrial size and myocardial extracellular volume fraction among persons living with and without HIV

**DOI:** 10.1186/s12872-022-02835-y

**Published:** 2022-09-03

**Authors:** Tess E. Peterson, Christian Landon, Sabina A. Haberlen, Fiona Bhondoekhan, Michael W. Plankey, Frank J. Palella, Damani A. Piggott, Joseph B. Margolick, Todd T. Brown, Wendy S. Post, Katherine C. Wu

**Affiliations:** 1 Division of Cardiology, Department of Medicine, Johns Hopkins University School of Medicine, Baltimore, MD USA; 2 Department of Epidemiology, Johns Hopkins Bloomberg School of Public Health, Baltimore, MD USA; 3 Department of Epidemiology, Brown University School of Public Health, Providence, RI USA; 4 Department of Medicine, Georgetown University Medical Center, Washington, DC USA; 5 Department of Medicine, Northwestern University Feinberg School of Medicine, Chicago, IL USA; 6 Division of Infectious Diseases, Department of Medicine, Johns Hopkins University School of Medicine, Baltimore, MD USA; 7 Department of Molecular Microbiology and Immunology, Johns Hopkins Bloomberg School of Public Health, Baltimore, MD USA; 8 Division of Endocrinology, Diabetes, and Metabolism, Department of Medicine, Johns Hopkins University School of Medicine, Baltimore, MD USA

**Keywords:** Human immunodeficiency virus, Inflammation, Myocardial disease, Extracellular volume fraction, Left atrial volume, Fibrosis

## Abstract

**Background:**

Infection with human immunodeficiency virus (HIV) is associated with higher risk for myocardial disease despite modern combination antiretroviral therapy (cART). Factors contributing to this excess risk, however, remain poorly characterized. We aimed to assess cross-sectional relationships between elevations of left atrial volume index (LAVI) and myocardial extracellular volume (ECV) fraction that have been reported in persons living with HIV and levels of circulating biomarkers of inflammation, fibrosis, and myocyte stretch among persons living with and without HIV (PLWH, PLWOH).

**Methods:**

Participants from three cohorts of PLWH and PLWOH underwent cardiovascular magnetic resonance imaging for measurement of LAVI and ECV. Levels of circulating proteins (IL-6, sCD14, galectin-3, NT-proBNP, GDF-15, TIMP-2, MMP-2, and hsTnI) were measured using immunoassays. Associations were assessed using logistic and linear regression, adjusting for demographics, substance use, and clinical characteristics.

**Results:**

Among 381 participants with and without HIV, median age (IQR) was 55.1 (51.2, 58.4) years, 28% were female, 69% were Black, and 46% were current smokers. Sixty-two percent were PLWH (*n* = 235), of whom 88% were receiving cART and 72% were virally suppressed. PLWH had higher levels of sCD14 (*p* = < 0.001), GDF-15 (*p* = < 0.001), and NT-proBNP (*p* = 0.03) compared to PLWOH, while levels of other biomarkers did not differ by HIV serostatus, including IL-6 (*p* = 0.84). Among PLWH, higher sCD14, GDF-15, and NT-proBNP were also associated with lower CD4 + cell count, and higher NT-proBNP was associated with detectable HIV viral load. NT-proBNP was associated with elevated LAVI (OR: 1.79 [95% CI: 1.31, 2.44]; *p* < 0.001) with no evidence of effect measure modification by HIV serostatus. Other associations between HIV-associated biomarkers and LAVI or ECV were small or imprecise.

**Conclusions:**

Our findings suggest that elevated levels of sCD14, GDF-15, and NT-proBNP among PLWH compared to PLWOH observed in the current cART era may only minimally reflect HIV-associated elevations in LAVI and ECV. Future studies of excess risk of myocardial disease among contemporary cohorts of PLWH should investigate mechanisms other than those connoted by the studied biomarkers.

**Supplementary Information:**

The online version contains supplementary material available at 10.1186/s12872-022-02835-y.

## Introduction

Combination antiretroviral therapy (cART) use among persons living with HIV (PLWH) has resulted in longer life expectancy and a shift in causes of morbidity and mortality from acquired immunodeficiency syndrome (AIDS)-defining conditions to chronic aging-related non-communicable diseases, such as cardiovascular disease (CVD) [1, 2]. Extensive evidence suggests that HIV infection in the current cART era is an independent risk factor for myocardial disease [3], including incident heart failure (HF) with both reduced (HFrEF) and preserved (HFpEF) ejection fraction [4–6] and sudden cardiac death [7, 8]. Factors contributing to this excess risk, however, remain poorly characterized.

Advanced HIV disease is associated with systolic dysfunction and dilated cardiomyopathy, but with the advent of effective cART came a transition from this phenotype to one characterized more by subclinical differences in cardiac structure and diastolic abnormalities which may ultimately contribute to higher risk of incident clinical HFpEF observed in the current treatment era [9, 10]. Among a contemporary population of cART-treated PLWH compared to persons living without HIV (PLWOH) in the same setting, we recently reported a higher left atrial volume index (LAVI) and myocardial extracellular volume (ECV) fraction—a measure of fibrosis, edema, or inflammation—using cardiac magnetic resonance (CMR) imaging [11], both of which may presage diastolic dysfunction and HFpEF. No other cardiac structural or functional characteristics differed significantly by HIV serostatus within that study population, and similar associations between HIV serostatus and both measures of myocardial fibrosis and diastolic abnormalities have been described in the United States, Europe, and Africa [12–18].

Contemporary data are needed to understand the pathogenesis of HIV-associated myocardial injury and remodeling. Several biomarkers have been studied for associations with cardiac structure and function among the general population, including those of inflammation, cardiac stress and injury, and extracellular matrix remodeling and fibrosis. However, these relationships have been understudied in the context of HIV infection, where they may differ due to immune dysregulation and chronically elevated systemic inflammation that persists even in the context of cART.

Inflammation is a hallmark of chronic HIV infection and is intimately involved in the pathology of tissue remodeling and fibrosis. When inflammatory responses to tissue insult are imbalanced, further tissue damage can occur via prolonged insult by the original injurious stimuli or persistent activation of inflammatory pathways [19]. Epidemiologic evidence also suggests higher circulating levels of inflammation and immune activation markers are associated with fibrosis across organ systems, including within the myocardium [16, 20, 21]. However, in the context of cART-treated HIV infection, the characterization of inflammatory markers that may contribute to myocardial injury and remodeling remains limited.

The objective of this study was to evaluate the cross-sectional relationships of biomarkers of inflammation, ventricular and cellular stress, tissue remodeling, and tissue injury with previously identified HIV-associated subclinical alterations in cardiac structure. We studied PLWH and PLWOH who were enrolled in one of three established cohort studies—the Multicenter AIDS Cohort Study (MACS), the Women’s Interagency HIV Study (WIHS), and the AIDS Linked to the IntraVenous Experience (ALIVE) study—and we focused our analyses to biomarkers and the CMR structural metrics of LAVI and ECV, which were associated with HIV serostatus within this study population.

## Methods

### Study population

Active participants of MACS, WIHS, and ALIVE—each described in detail previously—were recruited and consented into the current study for CMR imaging under identical, standardized protocols from 2015 to 2018 [11]. Briefly, the MACS is a prospective cohort study of men who have sex with men living with and without HIV infection across four cities in the United States; the WIHS is a prospective cohort of women living with and without HIV across ten US cities; and the ALIVE study is a community-based prospective cohort of persons with a history of injection drug use enrolled in Baltimore, MD. By nature of the parent cohort designs, sex at birth and substance use differed across cohorts—i.e., MACS participants were 100% male, WIHS participants were 100% female, and ALIVE participants had higher reported usage of opioids and stimulants. As previously reported, there were differences in other major factors across cohorts, such as race/ethnicity, with 47%, 5%, and 3% self-reported white participants in MACS, WIHS, and ALIVE, respectively [11]. Distributions of age, HIV seropositivity, and HIV-related factors such as cART use were similar across parent cohorts. Distributions of most important characteristics were similar between PWOH and PLWH—including age, sex, race/ethnicity, and traditional cardiovascular risk factors [11].

Participants of the current study were aged 40–70 years and actively enrolled in MACS, WIHS, or ALIVE at Chicago, Baltimore, or Washington, DC study sites. Exclusion criteria were any contraindications to CMR or contrast administration, including estimated glomerular filtration rate (eGFR) < 45 mL/min/1.73 m^2^, weight > 350 pounds, known claustrophobia, or known gadolinium contrast allergy.

### Clinical data

Under protocols of each of the included parent cohorts, participants underwent structured semi-annual study visits, which included standardized interviews, physical examinations, and plasma collection and storage. Data collected include demographics, education, smoking status, systolic and diastolic blood pressures, body mass index (BMI), fasting glucose; total, low-density lipoprotein (LDL), and high-density lipoprotein (HDL) cholesterol levels; serum creatinine, eGFR, hematocrit, and hepatitis C viral (HCV) infection status. Substance use variables were derived from data collected over the 5 years preceding CMR; any reported substance use during that time period is reported as use, and pack-years were calculated over this 5 year time period, not lifetime cumulative. In PLWH, measures of HIV disease activity included plasma HIV RNA concentrations (quantified down to 50 copies/mL, Roche ultrasensitive assay), current and nadir CD4 + T cell counts/µL, history of AIDS-defining malignancy or opportunistic infection, and use of cART.

Participants enrolled in the current study underwent a single visit between March 2015 and February 2018 where CMR was performed and a structured interview was administered, collecting further data on prescribed medications, history of cardiovascular disease, and substance use—including alcohol, marijuana, opiates, stimulants, prescribed or recreational use of erectile dysfunction agents, and prescribed or recreational use of nitrates.

### Cardiac magnetic resonance imaging

CMR was performed at Johns Hopkins Hospital (Baltimore, MD, USA) or Northwestern Memorial Hospital (Chicago, IL, USA) on 1.5T Siemens Avanto or Aera scanners (Erlangen, Germany) using a standardized protocol, described in detail previously and identical across these two sites [11]. Briefly, short- and long-axis cines (30 phases/cardiac cycle) were acquired using a steady-state free precession sequence; two-dimensional late gadolinium enhancement short- and long-axis images were acquired using an inversion-recovery fast gradient-echo sequence 15 min post-intravenous administration of 0.2 mmol/kg of gadobutrol contrast (Gadavist^®^, Bayer, Montville, NJ, USA); and T1 mapping was ascertained using a modified Look-Locker inversion (MOLLI) recovery sequence both before and after contrast administration. All CMR analyses were performed blinded to HIV serostatus and other participant characteristics using Segment v2.0 (http://segment.heiberg.se), Multimodality Tissue Tracking software (MTT; version 6.0, Toshiba, Japan), and MRmap version 1.2 (Charite University Medicine, Berlin, Germany).

LAVI and ECV fraction were selected as outcomes for the current analysis because they have been previously shown to differ by HIV serostatus in this study population [11]. Maximum LA volume was measured using the LA volume curve generated by the Simpson’s method from the four-chamber and two-chamber views and indexed by body surface area [22, 23]. Tracing of the LA borders excluded pulmonary veins and the left atrial appendage [22]. ECV fraction (%) was calculated as ECV = 100 × partition coefficient × [1 − hematocrit]), where the partition coefficient was derived from the slope of the linear relationship of 1/T1_myocardium_ veruss 1/T1_blood_ pre- and post-contrast [24].

### Protein biomarkers

Blood was drawn from participants concurrent with CMR, and samples were processed for serum and plasma on the same day using standard protocols and stored at − 80 °C until assays were performed. Biomarkers of inflammation (interleukin-6 [IL-6], soluble cluster of differentiation 14 [sCD14], galectin-3), ventricular and cellular stress (N-terminal prohormone of brain natriuretic peptide [NT-proBNP], growth differentiation factor-15 [GDF-15]), tissue remodeling (tissue inhibitor of metalloproteinase-2 [TIMP-2], matrix metalloproteinase-2 [MMP-2]) and tissue injury (high-sensitivity troponin I [hsTnI]) were measured in serum and plasma at central laboratories. Serum levels of IL-6 were measured by electrochemiluminescence (Meso Scale Discovery V-PLEX), and the following markers were measured by enzyme-linked immunosorbent assay: sCD14 (R&D Systems Quantikine), GDF-15 (R&D Systems Quantikine), TIMP-2 (R&D Systems Quantikine), MMP-2 (R&D Systems Quantikine), and NT-proBNP (Abbott Architect i2000sR) in serum, and galectin-3 (Abbott Architect i2000sR) and hsTnI (Abbott Architect i2000sR) in plasma.

### Statistics

Biomarkers were winsorized to their 97th percentiles to limit the effect of outliers in modeling procedures and standardized for interpretability. Cardiac phenotypes of interest were dichotomized at normative thresholds as previously described [11]. Specifically, elevated left atrial volume was defined as ≥ 40 mL/m^2^ following indexation by body surface area [25] and elevated ECV fraction was defined as ≥ 30% among women and ≥ 28% among men [26].

Participant characteristics, CMR imaging parameters (continuous and dichotomized LAVI and ECV fraction), and laboratory measures were summarized by median (interquartile range [IQR]) or proportion (count). The distributions of participant characteristics, including circulating biomarker levels and CMR parameters, were compared by HIV serostatus using the Wilcoxon rank-sum test for continuous variables and Pearson’s χ^2^ test for categorical variables. In order to minimize multiple testing, only biomarkers that were associated with HIV serostatus were evaluated in subsequent analyses.

Among PLWH, analyses were performed using multivariable linear regression to estimate the associations between measures of HIV disease severity and biomarkers that differed by HIV serostatus (identified in the previous analytic step), adjusting for age, sex, race/ethnicity, education, pack-years of smoking over the 5 years preceding CMR, and hazardous alcohol use defined by an Alcohol Use Disorders Identification Test (AUDIT) score > 8 at the time of CMR. For these analyses, biomarker responses were modeled on the natural log scale. Exponentiated *β* coefficients are therefore the ratios of the geometric mean biomarker concentrations among those exposed versus unexposed to the HIV clinical factor.

The cross-sectional associations between HIV-associated biomarkers (identified in the previous analytic step) and HIV-associated cardiac phenotypes (high LAVI and high ECV fraction) were estimated per standard deviation increment in biomarker concentration using multivariable logistic regression. These regression models were adjusted for HIV serostatus, age, sex, race/ethnicity, education level, and traditional cardiovascular risk factors—specifically history of cardiovascular disease, average continuous systolic blood pressure measured over 5 years preceding CMR, antihypertensive medication, dyslipidemia (use of lipid-lowering medication, fasting total cholesterol level ≥ 200 mg/dL, LDL-cholesterol level ≥ 130 mg/dL, HDL-cholesterol level < 40 mg/dL, or serum triglyceride level ≥ 150 mg/dL), diabetes (use of hypoglycemic medication or fasting serum glucose levels ≥ 126 mg/dL at the cohort study visit closest to the time of the CMR study; hemoglobin A1C level < 6.5% was used to exclude diabetes if a participant was not using hypoglycemic medication and fasting glucose levels were unavailable), pack-years of smoking over 5 years preceding CMR, and hazardous alcohol use at the time of CMR. We selected this model based on hypothesized confounders and did not adjust for parent cohort due to collinearity with sex, i.e., WIHS enrolled only women and MACS enrolled only men.

To assess heterogeneity of the associations between biomarkers and outcomes by HIV serostatus, we stratified analyses by HIV serostatus and tested a multiplicative interaction term for each biomarker (HIV × biomarker) in separate regression models. The stratified models included the same covariates as in the unstratified analyses, with the exception of cardiovascular disease history and hypertension medication due to small cell counts. We also assessed whether inclusion of biomarkers attenuated the association between HIV serostatus and either high ECV or high LAVI by comparing HIV effect estimates from multivariable models with and without individual outcome-associated biomarkers included as covariates. Finally, post-hoc sensitivity analyses were performed to evaluate the impact of HCV infection and additional recreational drug use covariates—specifically marijuana, opioid, stimulant, erectile dysfunction drug, and nitrate use. All analyses were conducted using Stata 16.1 and SAS 9.4 with a type I error rate of 0.05.

## Results

A total of 468 persons were initially enrolled across the three cohorts, of whom 381 had complete covariate data and either LAVI (*n* = 380) or ECV fraction (*n* = 319) acquired on CMR (Additional file 1: Figure S1). Demographic and clinical characteristics are presented in Table 1. Overall median age (IQR) was 55.1 (51.2, 58.4) years, 28% were female, 69% were Black, and 46% were current smokers. Sixty-two percent were PLWH, 88% of whom were receiving cART, and 72% of whom were virally suppressed. On average, PLWH had significantly higher levels of circulating sCD14 (*p* = < 0.001), GDF-15 (*p* =  < 0.001), and NT-proBNP (*p* = 0.03) compared to PLWOH; whereas there was no statistically significant difference by HIV serostatus in IL-6 (*p* = 0.84), galectin-3 (*p* = 0.13), TIMP-2 (*p* = 0.93), MMP-2 (*p* = 0.59), or hsTnI (*p* = 0.31) (Table 2). Only sCD14, GDF-15, and NT-proBNP were evaluated in subsequent analyses.

**Table 1:** Participant characteristics by HIV serostatus (*n* = 381)

Characteristic	Median [IQR] or % (*n*)		*p*-value^a^
	PLWOH (*n* = 146)	PLWH (*n* = 235)	
Parent cohort	–	–	0.28
MACS	68 (47%)	112 (48%)	
WIHS	22 (15%)	48 (20%)	
ALIVE	56 (38%)	75 (32%)	
*Cardiovascular magnetic resonance imaging metrics*			
Left atrial volume index, mL/m^2^	26.0 (21.4, 32.5)	27.7 (22.3, 35.7)	0.06
Left atrial volume index ≥ 40 mL/m^2^	8 (5%)	33 (14%)	0.01
ECV fraction, %	28.2 (25.9, 29.8)	28.7 (26.4, 30.9)	0.03
ECV fraction ≥ 30% women, ≥ 28% men	55 (44%)	108 (56%)	0.04
LV mass index, g/m^2^	60.9 (55.7, 66.7)	61.7 (56.2, 67.8)	0.51
LV end-diastolic volume index, mL/m^2^	64.3 (53.8, 72.7)	66.9 (55.8, 76.2)	0.20
LV end-systolic volume index, mL/m^2^	17.5 (13.3, 22.1)	18.2 (13.9, 22.7)	0.16
LV ejection fraction, %	73 (68, 76)	72 (68, 76)	0.31
*Demographics*			
Age, years	56 (52, 59)	55 (50, 58)	0.23
Female	37 (25%)	68 (29%)	0.45
Race/ethnicity	–	–	0.36
Black	95 (65%)	169 (72%)	
White	40 (27%)	53 (23%)	
Other	11 (8%)	13 (6%)	
Education	–	–	0.03
Less than high school	32 (22%)	67 (29%)	
Completed high school	45 (31%)	68 (29%)	
Some college	19 (13%)	47 (20%)	
College degree or higher	50 (34%)	53 (23%)	
Annual income > $10,000	79 (57%)	139 (62%)	0.35
*Substance use*			
Smoking status	–	–	0.09
Current	59 (40%)	116 (49%)	
Former	58 (40%)	68 (29%)	
Never	29 (20%)	51 (22%)	
Pack-months of smoking among current or former smokers^b^	11.8 (0.0, 32.9)	14.2 (2.0, 36.4)	0.02
AUDIT hazardous alcohol use	21 (14%)	25 (11%)	0.28
Marijuana use^b^	59 (40%)	106 (45%)	0.37
Opioid use^b^	47 (32%)	71 (30%)	0.68
Stimulant use^b^	52 (36%)	95 (40%)	0.35
Erectile dysfunction drug use^b^	23 (16%)	41 (17%)	0.67
Nitrate use^b^	22 (15%)	36 (15%)	0.95
*Clinical*			
Body mass index, kg/m^2^	27.1 (24.1, 31.0)	26.1 (23.3, 30.8)	0.08
Hypertension	79 (54%)	123 (52%)	0.74
Antihypertensive medication	50 (34%)	86 (37%)	0.64
Systolic blood pressure, mmHg^b^	128 (121, 135)	125 (119, 135)	0.10
Diastolic blood pressure, mmHg^b^	81 (76, 86)	79 (74, 85)	0.10
Dyslipidemia	85 (58%)	143 (61%)	0.61
Lipid-lowering medication	34 (23%)	58 (25%)	0.76
Total cholesterol, mg/dL	178 (149, 207)	172 (151, 195)	0.09
HDL-cholesterol, mg/dL	56 (46, 68)	51 (40, 63)	0.01
LDL-cholesterol, mg/dL	99 (78, 121)	93 (72, 115)	0.10
Diabetes	20 (14%)	30 (13%)	0.79
Fasting glucose, mg/dL	92 (84, 100)	91 (82, 100)	0.43
Diabetes medication	17 (12%)	23 (10%)	0.57
Creatinine, mg/dL	0.8 (0.7, 1.0)	0.9 (0.8, 1.1)	< 0.001
eGFR < 60 mL/min/m^2^	3 (2%)	11 (5%)	0.18
Hematocrit, %	40.8 (37.8, 43.4)	39.6 (36.8, 42.1)	0.02
Known cardiovascular disease	5 (3%)	9 (4%)	0.84
Hepatitis C virus infection	29 (20%)	56 (24%)	0.32
*HIV Disease Characteristics*			
HIV viral load < 50 RNA copies/mL	–	170 (72%)	
CD4 + cell count, cells/µL	–	605 (411, 842)	
CD4 + nadir, cells/µL	–	276 (151, 415)	
History of clinical AIDS	–	35 (22%)	
On antiretroviral therapy	–	206 (88%)	
Antiretroviral therapy duration, years	–	12.2 (4.8, 15.9)	
Protease inhibitor-based regimen	–	80 (39%)	
NNRTI-based regimen	–	66 (32%)	
Integrase strand inhibitor-based regimen	–	58 (28%)	

*IQR*, interquartile range; *PLWOH*, persons living without HIV; *PLWH*, persons living with HIV; *HDL*, high-density lipoprotein; *LDL*, low-density lipoprotein; *eGFR*, estimated glomerular filtration rate; *LAVI*, left atrial volume index; *ECV*, extracellular volume; *LV*, left ventricular; *NNRTI*, non-nucleoside reverse transcriptase inhibitor; *PI*, protease inhibitor

^a^Unadjusted *p*-values computed using Wilcoxon rank-sum and Pearson’s X^2^ for continuous and categorical characteristics, respectively

^b^Derived from data over 5 years preceding cardiac magnetic resonance imaging; mean value is reported for continuous variables and 'yes’ is reported for dichotomous variables with use indicated at any time over those 5 years

**Table 2:** Circulating biomarkers of inflammation, fibrosis, and myocyte stretch stratified by HIV serostatus (*n* = 381)

Biomarker	Median [IQR]		*p*-value^a^
	PLWOH (*n* = 146)	PLWH (*n* = 235)	
sCD14, ng/mL	1424 (1243, 1618)	1662 (1399, 1892)	< 0.001
IL-6, pg/mL	1.3 (0.8, 2.2)	1.4 (0.9, 2.1)	0.84
Galectin-3, ng/mL	15.3 (12.0, 17.5)	15.1 (12.4, 18.9)	0.13
GDF-15, pg/mL	761 (593, 1003)	1091 (794, 1442)	< 0.001
TIMP-2, ng/mL	85.9 (77.3, 94.9)	85.7 (77.7, 95.7)	0.93
MMP-2, ng/mL	213 (188, 242)	210 (187, 247)	0.59
NT-proBNP, pg/mL	45.9 (27.6, 79.5)	50.5 (32.4, 110.3)	0.03
hsTnI, ng/mL	2.8 (2.1, 4.9)	3.1 (2.3, 5.1)	0.31

*IQR*, interquartile range; *PLWOH*, persons living without HIV; *PLWH*, persons living with HIV; *sCD14*, soluble cluster of differentiation 14; *IL-6*, interleukin-6; *GDF-15*, growth differentiation factor 15; *TIMP-2*, tissue inhibitor of metalloproteinase 2; *MMP-2*, matrix metalloproteinase 2; *NT-proBNP*, N-terminal prohormone of brain natriuretic peptide; *hsTnI*, high sensitivity troponin I

^a^Unadjusted *p*-values computed using Wilcoxon rank-sum test

Among PLWH, some measures of greater HIV disease severity were associated with higher circulating concentrations of sCD14, GDF-15 and NT-proBNP, independent of demographics and substance use (Additional file 1: Table S1). Associations were observed between CD4 + cell count < 500 cells/µL (yes vs. no) and plasma concentration of sCD14 (mean percent difference in sCD14: 9% higher [95% CI: 2–16], *p* = 0.01; GDF-15: 15% higher [2–31], *p* = 0.03; and NT-proBNP: 30% higher [2–65], *p* = 0.03). Higher NT-proBNP was also associated with detectable HIV viral load (30% higher [1–68]; *p* = 0.05).

**Fig. 1:**
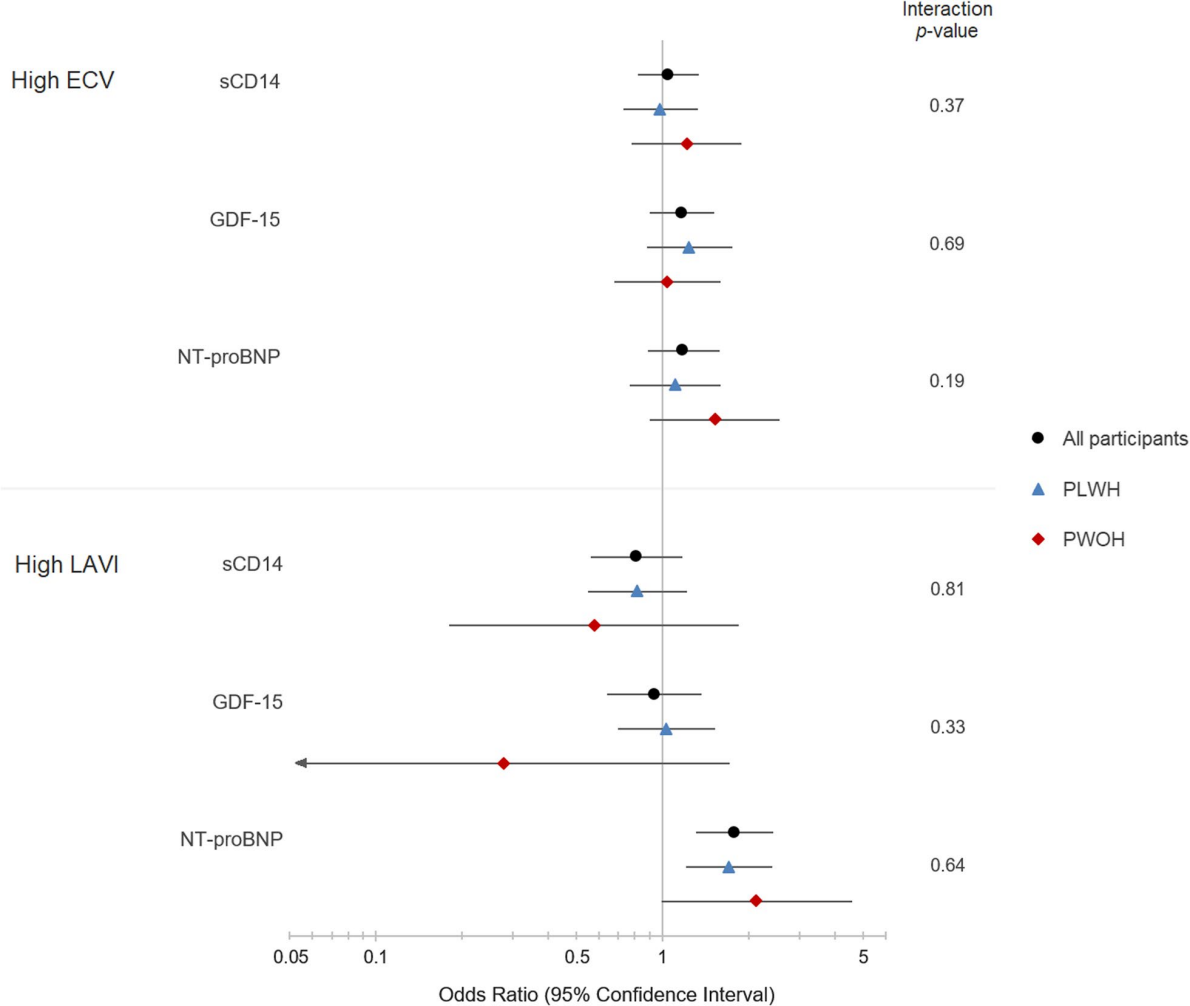
Adjusted associations between biomarkers of inflammation, fibrosis, and myocyte stretch that differed by HIV serostatus and cardiac structural characteristics (ECV and LAVI), overall and by HIV serostatus. Odds ratio and 95% confidence interval for high left atrial volume index (≥ 40 mL/m^2^) and high extracellular volume fraction (≥ 30% among women and ≥ 28% among men) are reported per standard deviation increment in biomarker, estimated using logistic regression adjusting for HIV serostatus, age, sex, race/ethnicity, education level, history of cardiovascular disease, systolic blood pressure, blood pressure-lowering therapy, dyslipidemia, diabetes, pack-years of smoking in 5 years preceding CMR, and hazardous alcohol use in 5 years preceding CMR. Null hypothesis is that odds ratio = 1. Interaction *p-*value is for a multiplicative interaction parameter, biomarker × HIV serostatus, used to assess effect measure modification. ECV = extracellular volume; LAVI = left atrial volume index; PLWH = persons living with HIV; PLWOH = persons living without HIV; sCD14 = soluble cluster of differentiation 14; GDF-15 = growth differentiation factor 15; NT-proBNP = N-terminal prohormone of brain natriuretic peptide

Figure 1 presents adjusted estimates of the association between biomarkers that differed by HIV serostatus in this study population—sCD14, NT-proBNP, and GDF-15—and high LAVI and ECV fraction [11], overall and by HIV serostatus. Among all participants, NT-proBNP was associated with elevated LAVI (odds ratio [OR]: 1.79 per SD increment in NT-proBNP [95% CI: 1.31–2.44]; *p* < 0.001), following adjustment for age, sex at birth, race/ethnicity, education, pack-years of smoking in 5 years preceding CMR, hazardous alcohol use, dyslipidemia, systolic blood pressure, blood pressure-lowering therapy, diabetes, history of CVD, and HIV infection. No HIV × biomarker interaction term was statistically significant, although subgroup estimates were imprecise due to rare outcomes and relatively small sample sizes (e.g., only 8 cases with elevated LAVI among those without HIV). Other associations between HIV-associated biomarkers and HIV-associated cardiac structural characteristics were either small or imprecise, both overall and within subgroups defined by HIV serostatus.

Adding NT-proBNP levels to adjusted models estimating the association between HIV and LAVI attenuated the magnitude of the HIV effect estimate slightly but did not alter the statistical significance of the association (OR without adjustment for NT-proBNP: 2.85 [95% CI: 1.26–6.45], *p* = 0.01; OR with this adjustment: 2.54 [95% CI: 1.11–5.81], *p* = 0.03; 11% decrease).

In sensitivity analyses, parsimonious models excluding non-significant covariates yielded biomarker parameter estimates with consistent magnitude and statistical significance with those in our primary models. Further adjustment for HCV infection and recreational drug use also yielded negligible differences in biomarker parameter estimates, as did further adjustment for detectable HIV viral load in subgroup analyses among PLWH.

## Discussion

Several lines of evidence now suggest PLWH in the current cART era have a higher prevalence of myocardial fibrosis, left atrial dilation, and diastolic abnormalities compared to PLWOH, including among participants within the current study [9–18, 27]. In this population of treated PLWH and PLWOH in the United States, positive HIV serostatus was associated with higher circulating levels of sCD14, GDF-15, and NT-proBNP, consistent with prior literature. When evaluating associations between levels of these three biomarkers and high LAVI or high ECV, there was evidence only of a relationship between NT-proBNP and LAVI, following adjustment for demographics and traditional CVD risk factors. Further, there was no evidence of modification of any effect estimates by HIV serostatus, assessed by HIV × biomarker interaction terms.

Contributors to excess risk of myocardial disease among PLWH are undoubtedly multifactorial, but immune dysfunction and persistent immune activation have been consistently implicated, even in the current cART era [28–34]. The interplay between inflammation, cardiac fibrosis, and cardiac stress is well-appreciated [19, 35], but remains poorly understood in HIV infection, where such relationships may be augmented or differ from those among the general population.

Higher circulating levels of sCD14, GDF-15, and NT-proBNP by HIV serostatus in this study are consistent with previous studies comparing cART-treated PLWH with PLWOH [16, 36–39]. CD14 is a pattern recognition receptor for ligands such as lipopolysaccharide [40]. It is integral to the activation of monocytes and macrophages and is involved in a variety of cellular processes, including host–pathogen interactions and cell differentiation [40]. A higher circulating level of sCD14 is a strong predictor of cardiovascular disease and mortality among both PLWOH and PLWH [41–43]. Consistent with our findings in both men and women, however, sCD14 was not associated with measures of myocardial fibrosis or diastolic dysfunction in a recent study among women living with well-controlled HIV in the United States [16]. The discrepant results in associations of sCD14 observed with clinical events but not subclinical myocardial disease or diastolic dysfunction may be due in part to difference in disease severity and potential differences in the role of monocyte and macrophage activation in atherosclerotic versus hypertensive cardiovascular pathologies [44]. For example, classical CD14^high^ monocytes are progenitors of M1-type pro-inflammatory and phagocytic macrophages directly involved in atherosclerotic plaque formation, while non-classical CD14^low^ monocytes are progenitors of M2-type macrophages, which may be pro-fibrotic in the context of chronic hemodynamic stress that can contribute to HF.

GDF-15 is a member of the transforming growth factor β superfamily involved in inflammation and cellular injury [45]. Elevated levels of GDF-15 have been strongly associated with all-cause mortality, incident HF, and poorer HF prognosis among the general population [46–49]. Higher GDF-15 has also been associated with subclinical cardiac structural and functional abnormalities among cART-treated PLWH—including higher LV mass index and lower LV ejection fraction—although conclusions on its association with diastolic dysfunction in particular have been inconsistent between study populations [38, 39]. To our knowledge, the association between GDF-15 and ECV fraction among PLWH has not previously been reported.

NT-proBNP is a powerful clinical biomarker used in diagnosis of HF, secreted in response to myocyte stretch due to increased ventricular preload, and elevated with both systolic and diastolic dysfunction [50]. Among PLWOH, elevated NT-proBNP is highly predictive of not only incident cardiovascular disease and mortality [51] but also subclinical cardiac remodeling, including myocardial fibrosis measured by ECV fraction [52, 53] and both cross-sectional and longitudinal increases in LAVI [54]. Among cART-treated PLWH, higher NT-proBNP has been cross-sectionally associated with higher left ventricular mass index, ECV fraction, and measures of both systolic and diastolic dysfunction [15, 39]. Results of the present study of LAVI are consistent with this literature but are not for ECV fraction, which may reflect lower absolute values and a narrower range of ECV in our study. The mild (11%) attenuation of the association between HIV infection and LAVI with addition of NT-proBNP as a model covariate suggests it may in part reflect HIV-associated differences in LAVI, but other HIV-related factors remain unmeasured and warrant further investigation.

We acknowledge limitations to this study. First, analyses were cross-sectional, so temporality and causation cannot be assessed. Second, sample size was relatively small, limiting our ability to detect small to moderate effect sizes or draw conclusions on effect measure modification by HIV serostatus. This is especially true for analyses of high LAVI, which was rare in our dataset. There are limitations inherent to the evaluated biomarkers, as well, specifically a lack of pathologic specificity. Even NT-proBNP, a well-established clinical biomarker used to rule out HF diagnosis, cannot reliably discriminate CVD pathologies [55] and may be elevated in extracardiac conditions such as renal insufficiency or systemic inflammation [56]. Interquartile ranges of some evaluated proteins were also relatively narrow, limiting prediction power. Additionally, while the distributions of demographic and key clinical characteristics among the participants in the current study are similar to that of the larger cohort previously published, we cannot rule out the possibility of selection bias due to analytic exclusions. Finally, multiple models were fit in this study, which may have led to type I errors.

In conclusion, our findings support prior literature establishing PLWH in the current cART era continue to have elevated levels of inflammation as well as markers of fibrosis and myocyte stress compared to PLWOH, specifically as measured by sCD14, GDF-15, and NT-proBNP. However, apart from an association between NT-proBNP and LAVI, the evaluated biomarkers do not well explain observed differences in LAVI and myocardial tissue abnormality by HIV serostatus. These results suggest mechanistic pathways other than those captured by the studied biomarkers should be investigated in future studies on excess risk of cardiovascular disease among contemporary cohorts of PLWH.

## Supplementary Information

**Additional file 1**: **Fig. S1**. Study participant flow diagram. **Table S1**. Adjusted associations between HIV clinical characteristics and biomarkers of inflammation, fibrosis, and myocyte stretch among persons living with HIV (*n* = 235).

## Data Availability

The datasets analyzed in the current study are available from the corresponding author on reasonable request.

## Abbreviations

AIDS: Acquired immunodeficiency syndrome
ALIVE: AIDS linked to the intra venous experience
AUDIT: Alcohol use disorders identification test
BMI: Body mass index
cART: Combination antiretroviral therapy
CMR: Cardiac magnetic resonance
CVD: Cardiovascular disease
ECV: Extracellular volume
eGFR: Estimated glomerular filtration rate
GDF-15: Growth differentiation factor-15
HCV: Hepatitis C viral
HDL: High-density lipoprotein
HF: Heart failure
HFpEF: Heart failure with preserved ejection fraction
HFrEF: Heart failure with reduced ejection fraction
hsTnI: High-sensitivity troponin I
IL-6: Interleukin-6
IQR: Interquartile range
LAVI: Left atrial volume index
LDL: Low-density lipoprotein
MACS: Multicenter AIDS cohort study
MMP-2: Matrix metalloproteinase-2
MOLLI: Modified look-locker inversion sequence
NT-proBNP: N-terminal prohormone of brain natriuretic peptide
OR: Odds ratio
PLWH: Persons living with HIV
PWOH: Persons living without HIV
sCD14: Soluble cluster of differentiation 14
TIMP-2: Tissue inhibitor of metalloproteinase-2
WIHS: Women’s interagency HIV study
