# Supplementary material for: Circulating biomarker correlates of left atrial size and myocardial extracellular volume fraction among persons living with and without HIV
Source: BMC Cardiovasc Disord. 2022 Sep 3;22:393. doi: 10.1186/s12872-022-02835-y (PMC9441072; doi:10.1186/s12872-022-02835-y)
Supplement: Supplementary file 1 — Additional file 1: Fig. S1. Study participant flow diagram. Table S1. Adjusted associations between HIV clinical characteristics and biomarkers of inflammation, fibrosis, and myocyte stretch among persons living with HIV (n = 235). [file 12872_2022_2835_MOESM1_ESM.pdf]

Circulating Biomarker Correlates of Left Atrial Size and Myocardial Fibrosis Among Persons Living With and Without HIV
*Supplemental Material*

SUPPLEMENTAL FIGURE 1. Study participant flow diagram.

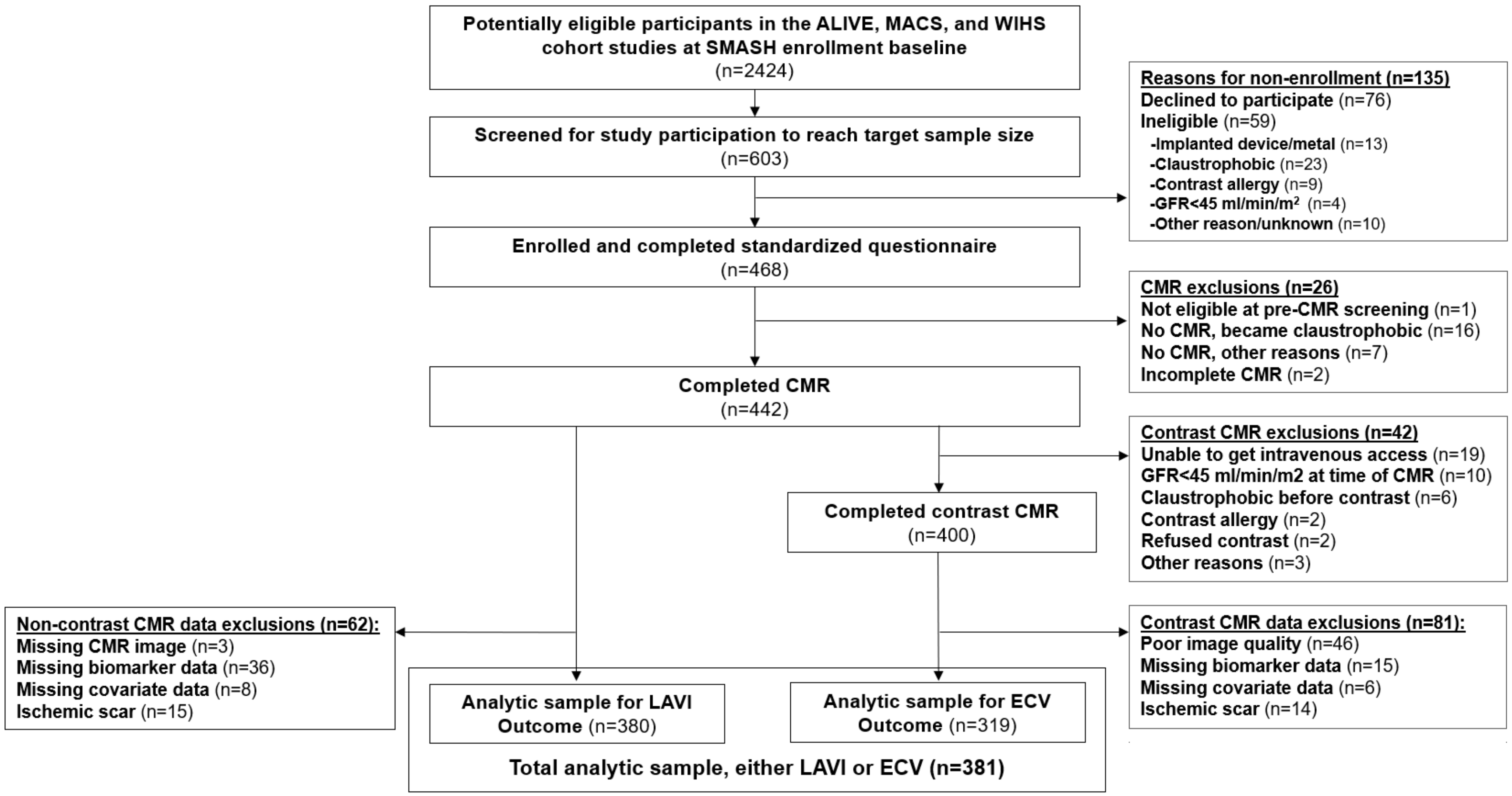


CMR=cardiovascular magnetic resonance imaging; GFR=estimated glomerular filtration rate; LAVI=left atrial volume index; ECV=extracellular volume.

SUPPLEMENTAL TABLE 1. Adjusted associations between HIV clinical characteristics and biomarkers of inflammation, fibrosis, and myocyte stretch among persons living with HIV (*n*=235).

| *HIV Characteristic* | *Exponentiated β Coefficient (95% CI)* [a] | | |
|---|---|---|---|
| | sCD14 | GDF-15 | NT-proBNP |
| Detectable HIV viral load | 0.99 (0.92, 1.07) | 1.02 (0.89, 1.17) | **1.30 (1.00, 1.68)** |
| CD4+ cell count <500 cells/µL | **1.09 (1.02, 1.16)** | **1.15 (1.02, 1.31)** | **1.30 (1.02, 1.65)** |
| Not receiving cART | 1.10 (1.00, 1.21) | 1.14 (0.95, 1.37) | 1.36 (0.97, 1.92) |
| History of clinical AIDS | 1.04 (0.95, 1.14) | 1.16 (0.97, 1.40) | 1.00 (0.73, 1.37) |

[a] Estimated using multivariable linear regression, adjusted for age, sex, race/ethnicity, education, pack-years of smoking in prior 5 years, and hazardous alcohol use in prior 5 years.

Biomarker outcomes were modeled on the natural log scale, so the exponentiated β coefficients presented are the ratios of the geometric mean biomarker concentrations among those exposed vs. unexposed to the HIV clinical factor. For example, individuals with detectable HIV viral load had 30% higher concentrations of NT-proBNP, on average, compared to those with undetectable viral load.

CI=confidence interval; sCD14=soluble CD14; GDF-15=growth differentiation factor 15; NT-proBNP=N-terminal prohormone of brain natriuretic peptide. **Bold** indicates *p*-value<0.05.
